# Supplementary material for: Interchromosomal interaction of homologous Stat92E alleles regulates transcriptional switch during stem-cell differentiation
Source: Nat Commun. 2022 Jul 9;13:3981. doi: 10.1038/s41467-022-31737-y (PMC9271046; doi:10.1038/s41467-022-31737-y)
Supplement: Supplementary file 1 — Supplementary information [file 41467_2022_31737_MOESM1_ESM.pdf]

Supplementary information for

Interchromosomal interaction of homologous *Stat92E* alleles regulates transcriptional switch during stem-cell differentiation

Matthew Antel[1], Romir Raj[1], Madona YG. Masoud[1], Ziwei Pan[2,3], Sheng Li [2,3], Barbara G. Mellone[4,5] and Mayu Inaba[1, *]

1. Department of Cell Biology, University of Connecticut Health Center, Farmington, CT 06030, USA
2. The Jackson Laboratory for Genomic Medicine, Farmington, CT, USA
3. The Department of Genetics and Genomic Sciences, The University of Connecticut Health Center, Farmington, CT, USA
4. Department of Molecular and Cell Biology, University of Connecticut, Storrs, CT 06269, USA
5. Institute for Systems Genomics, University of Connecticut, Storrs, CT 06269, USA

* Correspondence: inaba@uchc.edu

Contents;

Supplementary Figures (1-6) and legends

Legends for Supplementary Data (1, 2)

Description for Source Data

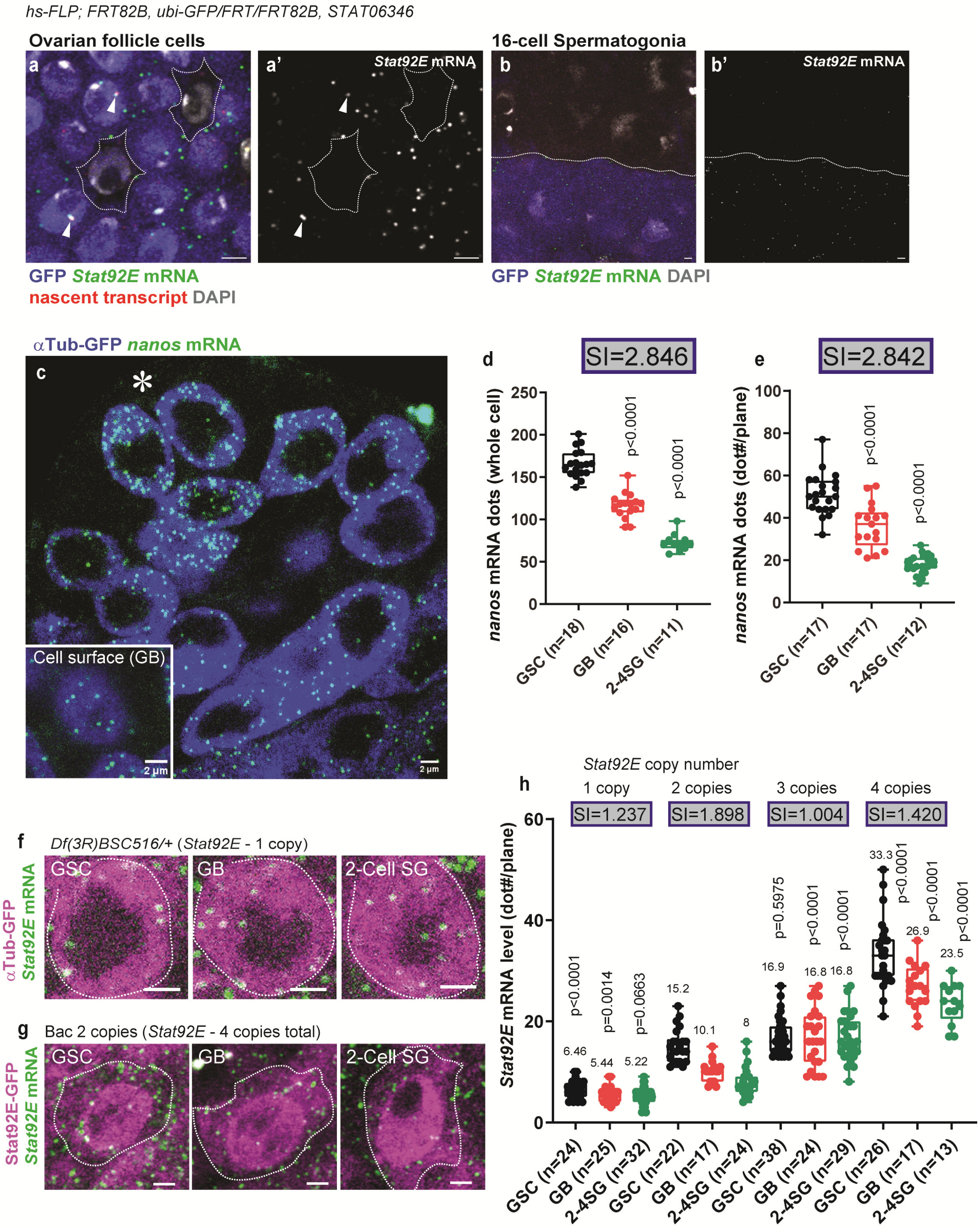
hs-FLP; FRT82B, ubi-GFP/FRT/FRT82B, STAT06346
Ovarian follicle cells
a
a'
Stat92E mRNA
16-cell Spermatogonia
b
b'
Stat92E mRNA
GFP Stat92E mRNA
nascent transcript DAPI
GFP Stat92E mRNA DAPI
αTub-GFP nanos mRNA
c
Cell surface (GB)
2 µm
2 µm
d
SI=2.846
nanos mRNA dots (whole cell)
250
200
150
100
50
0
p<0.0001
p<0.0001
GSC (n=18)
GB (n=16)
2-4SG (n=11)
e
SI=2.842
nanos mRNA dots (dot#/plane)
100
80
60
40
20
0
p<0.0001
p<0.0001
GSC (n=17)
GB (n=17)
2-4SG (n=12)
f
Df(3R)BSC516/+ (Stat92E - 1 copy)
αTub-GFP
Stat92E mRNA
GSC
GB
2-Cell SG
g
Bac 2 copies (Stat92E - 4 copies total)
Stat92E-GFP
Stat92E mRNA
GSC
GB
2-Cell SG
h
Stat92E copy number
1 copy
2 copies
3 copies
4 copies
SI=1.237
SI=1.898
SI=1.004
SI=1.420
Stat92E mRNA level (dot#/plane)
60
40
20
0
p<0.0001
p=0.0014
p=0.0663
p=0.5975
p<0.0001
p<0.0001
p<0.0001
p<0.0001
p<0.0001
6.46
5.44
5.22
15.2
10.1
8
16.9
16.8
16.8
33.3
26.9
23.5
GSC (n=24)
GB (n=25)
2-4SG (n=32)
GSC (n=22)
GB (n=17)
2-4SG (n=24)
GSC (n=38)
GB (n=24)
2-4SG (n=29)
GSC (n=26)
GB (n=17)
2-4SG (n=13)

Supplementary Figure 1.

Quantification of mRNA levels using smFISH.

a, b) Validation of *Stat92E* FISH probe using $STAT^{06346}$ clone (GFP negative). In **a**, $STAT^{06346}$ clone ovarian follicle cells are encircled by white dotted lines. Arrowheads indicate nascent transcript. In **b**, a white broken line divides boundary of $STAT^{06346}$ clone (upper half) and non-clone cells (lower half). **c**) A Representative image of smFISH against *nanos* exon probe. Inset shows surface view of a GB. An asterisk indicates the hub. **d, e**) Quantification of *nanos* mRNA. Y axis values are the number of smFISH dots present in an entire cell (whole cell in **C**) or a middle plane of the cell (dot#/plane in **e**). **f, g**) Representative smFISH images visualizing the *Stat92E* mRNA (green) using indicated genotypes. Germ cells are visualized by nos>αTubulin-GFP in **f** (magenta) or Stat92E-GFP in **g** (magenta) and encircled by white dotted lines. **h**) Quantification of *Stat92E* mRNA in cells with indicated copy number of *Stat92E* alleles. Genotypes used were; 1copy: *Stat92E Df/+* (*Df(3R)BSC516*), 2 copies: wild type (*yw*), 3 copies: *Stat92E BacTg/+* (*Bac VK00037*) in wild type background and 4 copies: *BacTg/BacTg* in wild type background. Y axis values are the number of smFISH dots. Average values are shown on the top of each column.

The adjusted p-values were calculated by one-way anova with Dunnett's multiple comparisons for comparing each dataset with GSC data in **d** and **e**, and one way anova with Šidák's multiple comparisons for comparing datasets with wild type (2 copies) data in **h**. Box plots show 25–75% (box), median (band inside) and minimum to maximum (whiskers) with all data points. All plotted data points are provided in Source Data. Number of scored cells, which are randomly chosen from at least 10 testes for each experiments, is shown for each data point. All scale bars represent 2 μm.

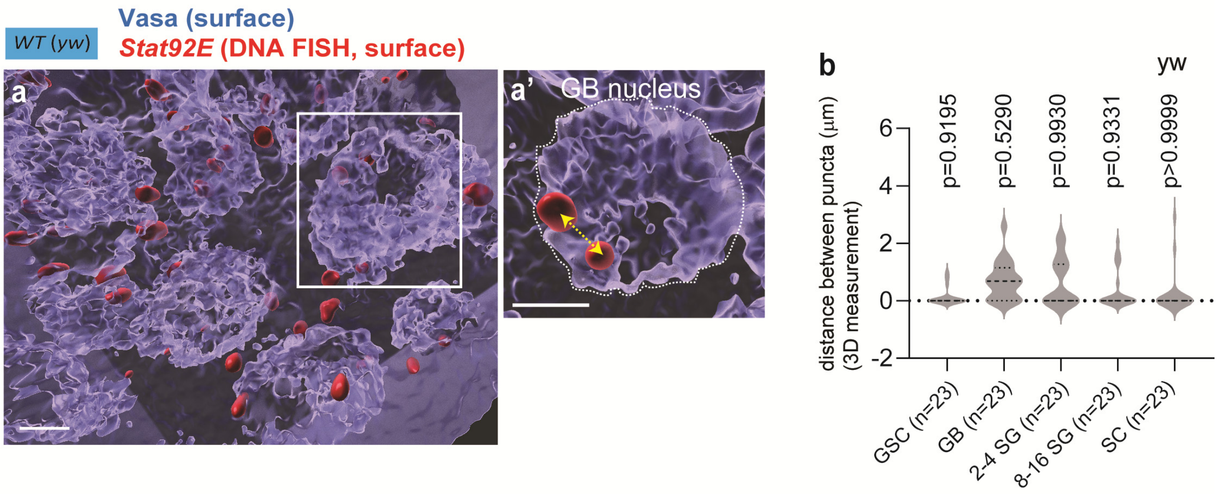

Supplementary Figure 2.

Measurement of distances between two Stat92E loci on homologous chromosomes.

a) To measure distances between DNA FISH or nascent transcript puncta, we imaged z-stack of entire testis tip with optimized interval (0.5μm to 1μm). First, we judged whether a cell has single punctate paired or two separate puncta unpaired pattern. For all paired cases in which punctate appears as a single spot, we plotted zero on each violin plot. When two signals were located in different z-stacks, we calculated the distance using a following equation by Microsoft Excel and plotted values on each violin plot.

$$\mathbf{D} = \sqrt[2]{x^2 + z^2} \tag{1}$$

where **D** is actual distance between punctae, x is a measured distance on a single plane and z is a z-distance. Paired punctae had approximately twice as much intensity as “unpaired” punctae (see Fig. 5e for quantification).

To confirm whether results obtained by our measurements are accurately reflecting three-dimensional (3D) distances of homologous loci, we performed 3D measurements for *Stat92E* DNA FISH signal in wild type (*yw*) sample using Imaris software by selecting two spots locating on different z-stacks. A shows a 3D rendering image of a wild type sample used for 3D measurement. A GB in a white square in A is shown from different angles in A’. Surface reconstruction of Vasa signal (purple) depicts the shape of nucleus where Vasa is negative. *Stat92E* DNA FISH signal is shown in red. A yellow arrow indicates two *Stat92E* alleles. **b**)

Violin plots showing the measured 3D distances between puncta of *Stat92E* DNA FISH in wild type (*yw*) fly testes using Imaris software (measurements were performed by using same image sets used for Fig. 2c).

Violin plots show KDE and quantile lines and the width of each curve corresponds with the frequency of data points. The adjusted p-values were calculated by one-way anova with Šidák's multiple comparisons for comparing datasets with wild type (yw) data shown in Fig. 2c. Plotted data points are provided in Source Data. Number of scored cells, which are randomly chosen from at least 10 testes for each experiments, is shown for each data point.

All scale bars represent 2 μm.

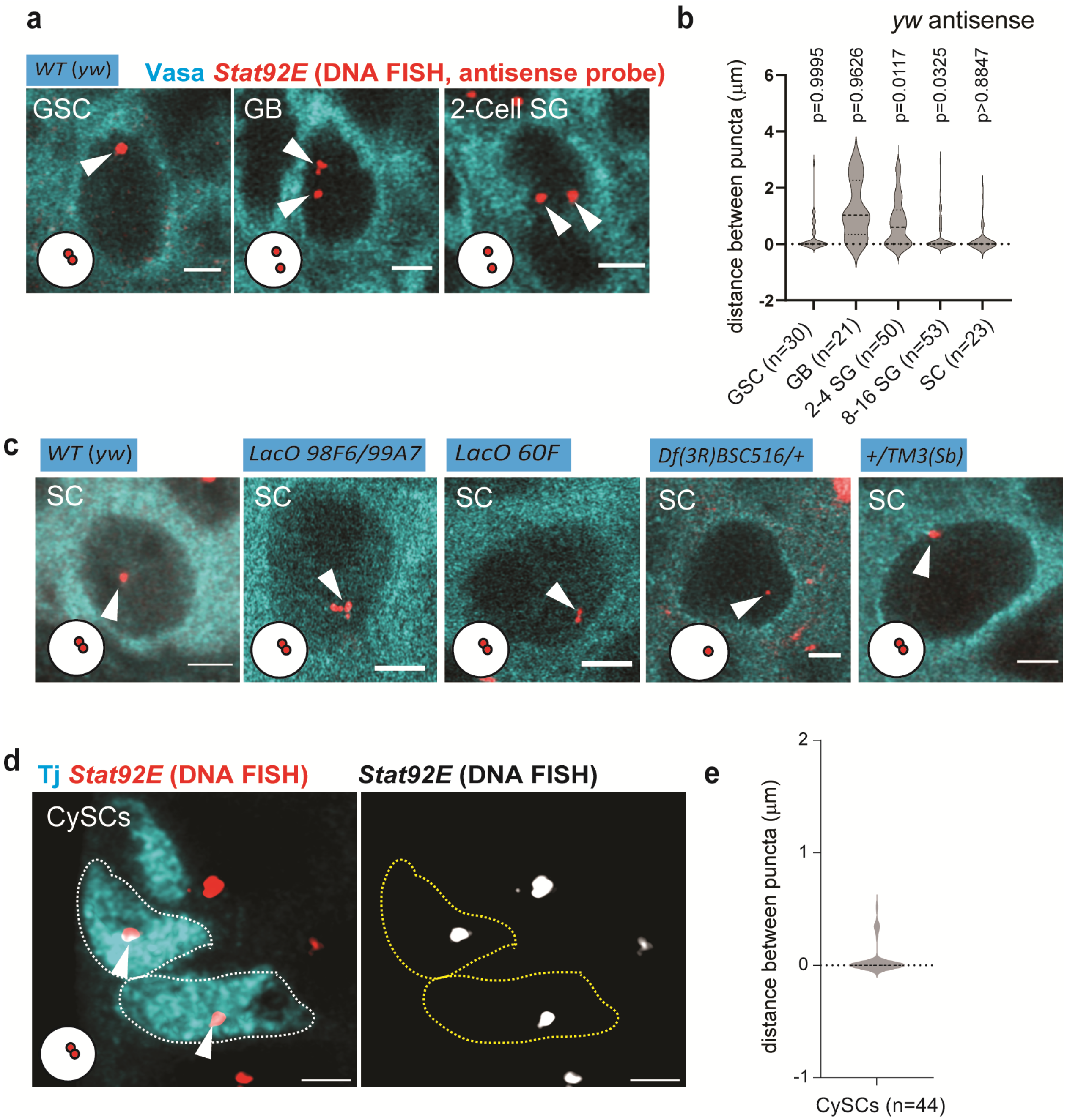

Supplementary Figure 3.

***Stat92E* Pairing is stage-, locus- and cell type-specifically regulated.**

a) Representative images of DNA FISH targeting the antisense strand of the *Stat92E* locus (red, pointed by white arrowheads) at the indicated stages of germ cell development in wild type (*yw*) fly testes. **b**) Violin plots showing the distance between puncta of DNA FISH. **c**) Representative images of DNA FISH targeting *Stat92E* locus (red, pointed by white arrowheads) of SCs in the indicated genotypes. Germ cells were visualized by Vasa staining (cyan). **d**) Representative

images of DNA FISH targeting *Stat92E* locus (red, pointed by white arrowheads) of CySCs (encircled by broken lines) in the wild type (*yw*). CySCs were visualized by staining of a CySC marker, traffic jam (Tj, cyan). **e**) A violin plot showing the distance between puncta of DNA FISH in CySCs in the wild type sample (*yw*). For all measurement of distances between puncta of DNA FISH, cells with more than three puncta were omitted from scoring. Diagrams in the corner of images in **a** and **c** represent the pairing states of each condition. All scale bars represent 2 μm. Each data point was obtained from at least two independent experiments. At least 10 testes were used for each experiment.

Violin plots show KDE and quantile lines and the width of each curve corresponds with the frequency of data points. The adjusted p-values were calculated by one-way anova with Šidák's multiple comparisons for comparing datasets with wild type (yw, sense probe) data shown in Fig. 2c. Plotted data points are provided in Source Data. Number of scored cells, which are randomly chosen from at least 10 testes for each experiments, is shown for each data point.

All scale bars represent 2 μm.

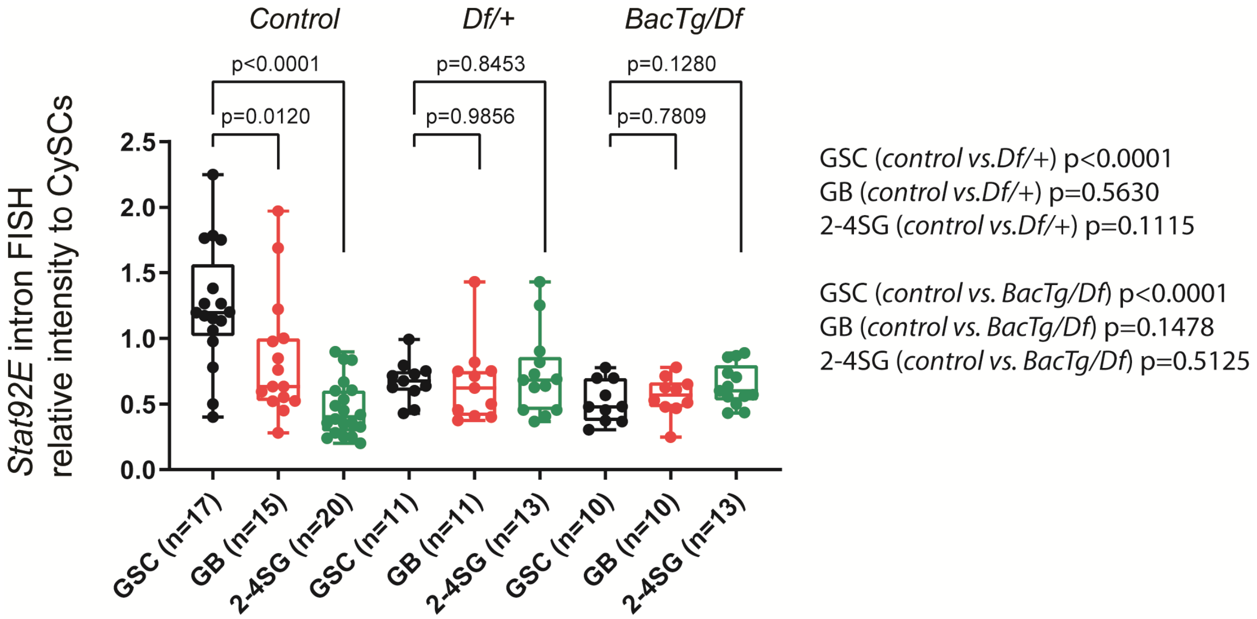

Supplementary Figure 4.

Compromised *Stat92E* pairing affects silencing of *Stat92E* nascent transcript.

Quantification of the level of *Stat92E* nascent transcript throughout differentiation of indicated genotypes. Measured intensities of paired cases were divided by two to represent the amount of nascent transcript /allele. Control (*nos>αTub-GFP*), heterozygous of *Stat92E* deficiency line, *Df(3R)BSC516* (*Df/+*) and *BacTg/Df* (see main text for this genotype) were used. Y axis indicates the average fluorescence intensity of nascent transcript signal in germ cells divided by randomly selected CySCs' appearing within the same image plane.

The adjusted p-values were calculated by one-way anova with Dunnett's multiple comparisons for comparing each stage with GSC data, or with Šidák's multiple comparisons for comparing between genotypes (listed right). Box plots show 25–75% (box), median (band inside) and minimum to maximum (whiskers) with all data points. All plotted data points are provided in Source Data. Number of scored cells, which are randomly chosen from at least 10 testes for each experiments, is shown for each data point.

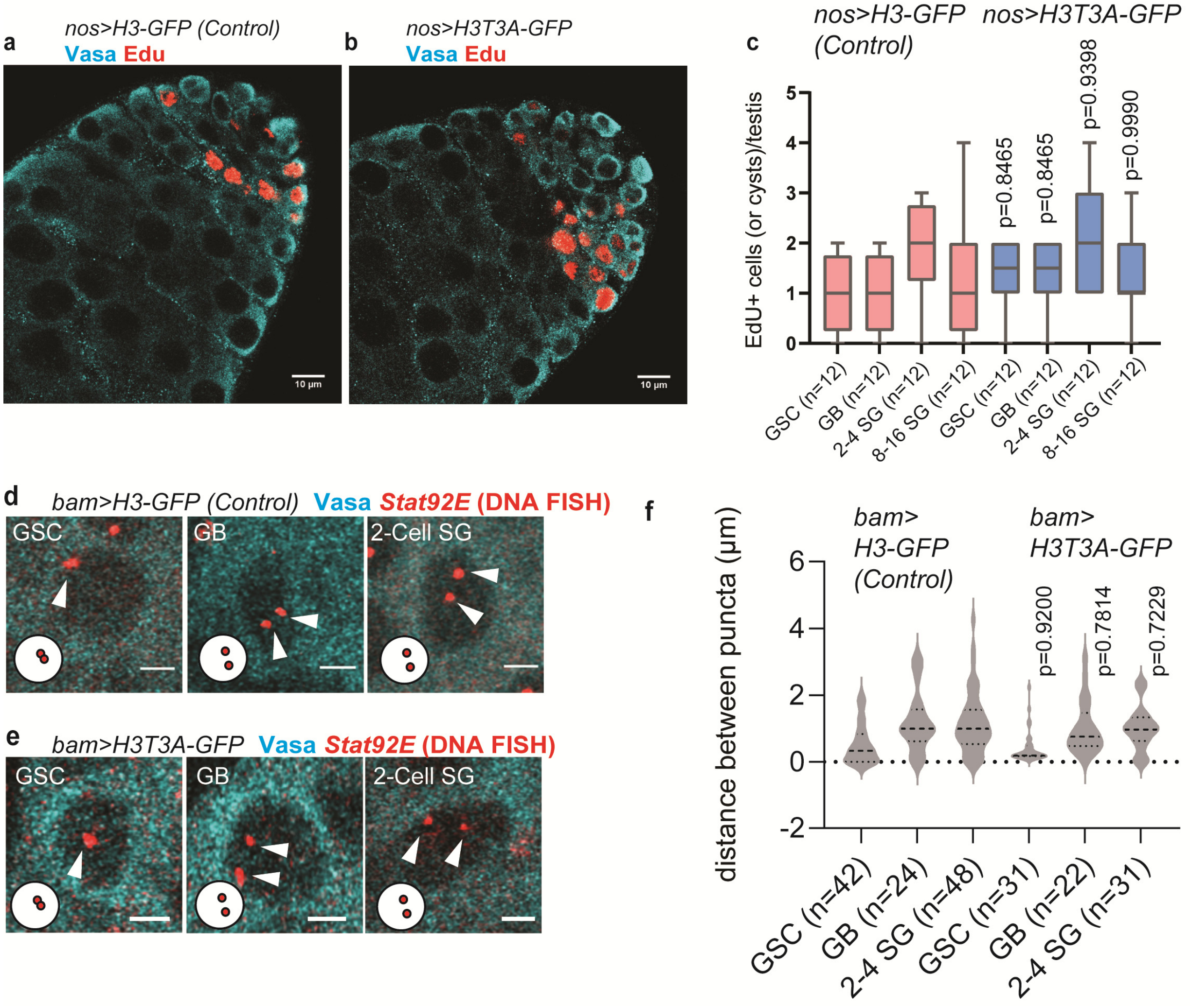

Supplementary Figure 5.

Changes in *Stat92E* pairing is preprogrammed by the asymmetric histone inheritance in GSC.

a, b) Representative images of EdU (red) incorporation in testes expressing histone H3-GFP (control, **a**) or histone H3T3A-GFP (**b**) under the control of the *nosGal4* driver. Scale bars represent 10 µm. **c**) Graph of quantified EdU positive germ cells (or SG cysts) per testis at the indicated stages of germ cell development from histone H3-GFP (control) or histone H3T3A-GFP expressing testes under the *nosGal4* driver. Because 2, 4, 8, or 16-cell SG cysts are typically synchronized to enter S-phase, we counted any EdU positive cysts as “one” for better comparison between genotypes**.** The sample size n represents the number of testes scored.

Box plots show 25–75% (box), median (band inside) and minimum to maximum (whiskers) with all data points.

d, **e**) Representative images of DNA FISH targeting the *Stat92E* locus (red, pointed by white arrowheads) at the indicated stages of germ cell development from histone H3-GFP (**d**) or histone H3T3A (**e**) expressing testes under the *bamGal4* driver. **f**) Violin plots of the distances between homologous *Stat92E* regions at the indicated stages of germ cell development from indicated genotypes. Violin plots show KDE and quantile lines and the width of each curve corresponds with the frequency of data points. Number of scored cells, which are randomly chosen from at least 10 testes for each experiments, is shown for each data point.

Scale bars in **a**, **b** represent 10 μm, scale bars in **d** and **e** represent 2 μm.

The adjusted p-values were calculated by one-way anova with Šidák's multiple comparisons for comparing with control data. Plotted data points are provided in Source Data.

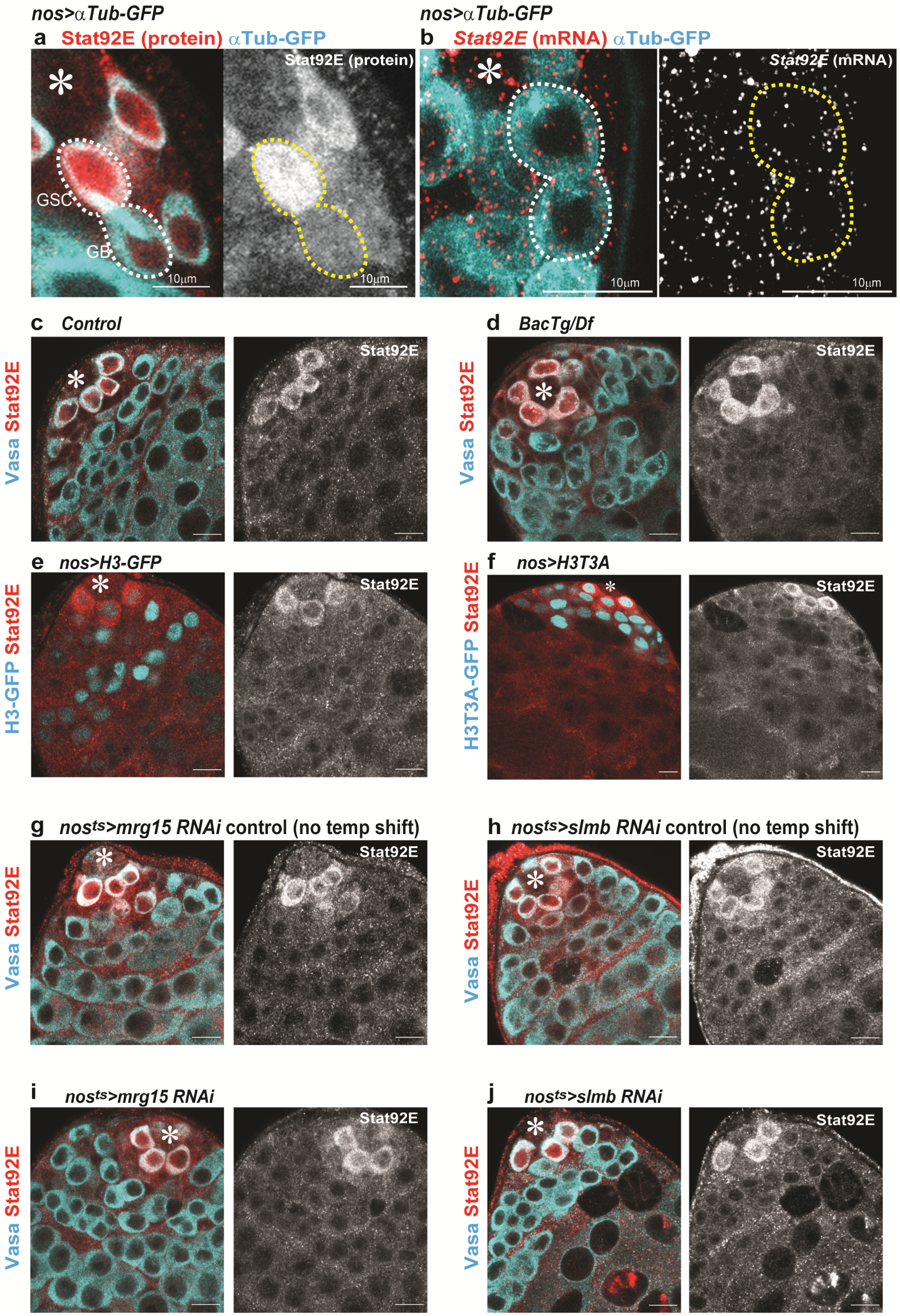

nos>αTub-GFP
a Stat92E (protein) αTub-GFP
Stat92E (protein)
GSC
GB
10μm
nos>αTub-GFP
b Stat92E (mRNA) αTub-GFP
Stat92E (mRNA)
10μm
c Control
Vasa Stat92E
Stat92E
d BacTg/Df
Vasa Stat92E
Stat92E
e nos>H3-GFP
H3-GFP Stat92E
Stat92E
f nos>H3T3A
H3T3A-GFP Stat92E
Stat92E
g nosts>mrg15 RNAi control (no temp shift)
Vasa Stat92E
Stat92E
h nosts>slmb RNAi control (no temp shift)
Vasa Stat92E
Stat92E
i nosts>mrg15 RNAi
Vasa Stat92E
Stat92E
j nosts>slmb RNAi
Vasa Stat92E
Stat92E

Supplementary Figure 6.

Defects in *Stat92E* pairing do not affect pattern of Stat92E protein expression.

a) Representative image of immunofluorescence staining of Stat92E protein (red) in a GSC-GB pair. **b**) Representative image of RNA FISH of *Stat92E* mRNA (red) in a GSC-GB pair. In **a** and **b**, Germ cells were visualized by expressing αTubulin-GFP (cyan) under the control of *nosGal4* driver. GSC-GB pairs are encircled by dotted lines.

c-j) Representative images of immunofluorescence staining of Stat92E protein (red) and Vasa (cyan) of indicated genotypes. Temperature shift (temp shift) was performed in 29 degrees for 5-7 days. Asterisks indicate the hub. All scale bars represent 10μm**.**
