# Supplementary material for: Interchromosomal interaction of homologous Stat92E alleles regulates transcriptional switch during stem-cell differentiation
Source: Nat Commun. 2022 Jul 9;13:3981. doi: 10.1038/s41467-022-31737-y (PMC9271046; doi:10.1038/s41467-022-31737-y)
Supplement: Supplementary file 3 — Description of additional Supplementary File [file 41467_2022_31737_MOESM3_ESM.pdf]

Descriptions of Additional Supplementary Data Files

Supplementary Data 1: Listed are the sequences of oligonucleotide used for the exon and intron probes of the Stat92E RNA FISH. The intron probe is conjugated with Quasar 570 dye, and the exon probe is conjugated with Quasar 670.

Supplementary Data 2: Listed are the sequences of oligonucleotide used for OligoPaint probe targeting the Stat92E locus. The sequences were generated using PaintSHOP online software (see Methods for details on OligoPaint probe production).
