# Supplementary material for: Interchromosomal interaction of homologous Stat92E alleles regulates transcriptional switch during stem-cell differentiation
Source: Nat Commun. 2022 Jul 9;13:3981. doi: 10.1038/s41467-022-31737-y (PMC9271046; doi:10.1038/s41467-022-31737-y)
Supplement: Supplementary file 6 — Reporting Summary [file 41467_2022_31737_MOESM6_ESM.pdf]

nature portfolio

Corresponding author(s): Mayu Inaba

Last updated by author(s): 06/2/2022

Reporting Summary

Nature Portfolio wishes to improve the reproducibility of the work that we publish. This form provides structure for consistency and transparency in reporting. For further information on Nature Portfolio policies, see our Editorial Policies and the Editorial Policy Checklist.

Statistics

For all statistical analyses, confirm that the following items are present in the figure legend, table legend, main text, or Methods section.

n/a	Confirmed	
☐	☒	The exact sample size (*n*) for each experimental group/condition, given as a discrete number and unit of measurement
☐	☒	A statement on whether measurements were taken from distinct samples or whether the same sample was measured repeatedly
☐	☒	The statistical test(s) used AND whether they are one- or two-sided<br>*Only common tests should be described solely by name; describe more complex techniques in the Methods section.*
☒	☐	A description of all covariates tested
☐	☒	A description of any assumptions or corrections, such as tests of normality and adjustment for multiple comparisons
☐	☒	A full description of the statistical parameters including central tendency (e.g. means) or other basic estimates (e.g. regression coefficient) AND variation (e.g. standard deviation) or associated estimates of uncertainty (e.g. confidence intervals)
☐	☒	For null hypothesis testing, the test statistic (e.g. *F*, *t*, *r*) with confidence intervals, effect sizes, degrees of freedom and *P* value noted<br>*Give P values as exact values whenever suitable.*
☒	☐	For Bayesian analysis, information on the choice of priors and Markov chain Monte Carlo settings
☒	☐	For hierarchical and complex designs, identification of the appropriate level for tests and full reporting of outcomes
☒	☐	Estimates of effect sizes (e.g. Cohen's *d*, Pearson's *r*), indicating how they were calculated

Our web collection on statistics for biologists contains articles on many of the points above.

Software and code

Policy information about availability of computer code

Data collection	Confocal images were collected by a Zeiss confocal LSM800 airy scan with a 63× oil immersion objective (NA=1.4) using Zen software (version Zen Blue 2.1).
Data analysis	Confocal images were analyzed using ImageJ and Fiji software (version 2.1.0) and Imaris 9.5. Statistical analysis and figures were generated using GraphPad Prism software (version 9.2.0) and Microsoft Excel.

For manuscripts utilizing custom algorithms or software that are central to the research but not yet described in published literature, software must be made available to editors and reviewers. We strongly encourage code deposition in a community repository (e.g. GitHub). See the Nature Portfolio guidelines for submitting code & software for further information.

Data

Policy information about availability of data

All manuscripts must include a data availability statement. This statement should provide the following information, where applicable:

- Accession codes, unique identifiers, or web links for publicly available datasets
- A description of any restrictions on data availability
- For clinical datasets or third party data, please ensure that the statement adheres to our policy

Complete data is available in the main article text and figures, supplementary figures. Source data are provided with this paper. Original images used for quantifications are deposited to the BioStudies database, accession number S-BSST829.

Field-specific reporting

Please select the one below that is the best fit for your research. If you are not sure, read the appropriate sections before making your selection.

- [x] Life sciences
- [ ] Behavioural & social sciences
- [ ] Ecological, evolutionary & environmental sciences

For a reference copy of the document with all sections, see nature.com/documents/nr-reporting-summary-flat.pdf

Life sciences study design

All studies must disclose on these points even when the disclosure is negative.

Sample size	No statistical test was performed to predetermine sample size. All experiments were performed using a minimum of 10 testes, and experiments were repeated 2 times for Fig. 3c and Fig. S5c, or 3 times for other figures. In our experience, this gives accurate representation and variability between replicates was not dramatically changed in any group tested.
Data exclusions	No data were excluded.
Replication	All data were successfully reproduced with replicates for each experiment.
Randomization	Experimental groups were chosen based on genotypes, and treatments (temperature shift). All animals selected for processing, imaging, and analysis were chosen at random with no bias.
Blinding	Researchers were not blinded to data collection or analysis as blinding is not standard in the field. Different genotypes were used alongside multiple controls, and treatment such as temperature shifting is easily detectable.

Reporting for specific materials, systems and methods

We require information from authors about some types of materials, experimental systems and methods used in many studies. Here, indicate whether each material, system or method listed is relevant to your study. If you are not sure if a list item applies to your research, read the appropriate section before selecting a response.

Materials & experimental systems

n/a	Involved in the study	
	x	Antibodies
x		Eukaryotic cell lines
x		Palaeontology and archaeology
	x	Animals and other organisms
x		Human research participants
x		Clinical data
x		Dual use research of concern

Methods

n/a	Involved in the study	
x		ChIP-seq
x		Flow cytometry
x		MRI-based neuroimaging

Antibodies

Antibodies used	The primary antibodies used were: guinea pig anti-Stat92E (Inaba M, Buszczak M, Yamashita YM. Nature. 2015;523(7560):329-332. doi:10.1038/nature14602, 1:2000), rat anti-Vasa (DSHB, anti-vasa/AB_760351, developed by A. Spradling and D. Williams, 1:20), and rabbit anti-Vasa (d-260, Santa Cruz Biotechnology, Santa Cruz, CA, 1:200), and guinea pig anti-traffic jam (Li MA, Alls JD, Avancini RM, Koo K, Godt D. Nat Cell Biol. 2003;5(11):994-1000. doi:10.1038/ncb1058, 1:4000).<br><br>The secondary antibodies used were Goat Anti-Rabbit IgG H&L (Alexa Fluor 488, Abcam, ab175652), Goat Anti-Rabbit IgG H&L (Alexa Fluor 647, Abcam, ab150079), Goat Rat IgG H&L (Alexa Fluor 488, Abcam, ab150157), Goat Rat IgG H&L (Alexa Fluor 647, Abcam, ab150159), and Goat Anti-Guinea Pig IgG H&L (Alexa Fluor 647, Abcam, ab150187). All secondary antibodies were used at a dilution of 1:400)
Validation	guinea pig anti-Stat92E: please see Inaba M, Buszczak M, Yamashita YM. Nature. 2015;523(7560):329-332. doi:10.1038/nature14602<br><br>rat anti-Vasa: please see Song Y, Lu B. Genes Dev. 2011;25(24):2644-2658. doi:10.1101/gad.171959.111 and also https://dshb.biology.uiowa.edu/anti-vasa<br><br>rabbit anti-Vasa: please see Yang L, Duan R, Chen D, Wang J, Chen D, Jin P. Hum Mol Genet. 2007;16(15):1814-1820. doi:10.1093/hmg/ddm129 and also https://www.scbt.com/p/vasa-antibody-d-260<br><br>guinea pig anti-traffic jam: please see Li MA, Alls JD, Avancini RM, Koo K, Godt D. Nat Cell Biol. 2003;5(11):994-1000. doi:10.1038/ncb1058

Animals and other organisms

Policy information about studies involving animals; ARRIVE guidelines recommended for reporting animal research

Laboratory animals	Various strains of Drosophila melanogaster were used in this study, obtained from Bloomington Drosophila Stock Center or from members of the Drosophila research community. New transgenic flies used in this study were generated by BestGene Inc. Details on each strain used can be found in the Methods section under Fly husbandry and strains.
Wild animals	No wild animals were used in this study, only laboratory strains of Drosophila melanogaster.
Field-collected samples	No field collection samples were used in this study.
Ethics oversight	No ethical approval was required for this study because Drosophila melanogaster use is not regulated by The Animals (Scientific Procedures) Act of 1986.

Note that full information on the approval of the study protocol must also be provided in the manuscript.
